# *Leishmaniasis* prevalence, awareness and control in Saudi Arabia

**DOI:** 10.4314/ahs.v22i3.68

**Published:** 2022-09

**Authors:** Taha A Kumosani, Turkyah J Al-Bogami, Elie K Barbour, Sharifah H Alshehri, Soonham S Yaghmoor, Nouf A Alshareef, Khalid M El-Say, Said S Moselhy

**Affiliations:** 1 Biochemistry Department, Faculty of science, King Abdulaziz University, Jeddah, Kingdom of Saudi Arabia; 2 Experimental Biochemistry Unit, King Fahd Medical Research Center, King Abdulaziz University P.O.Box 21424, Jeddah, Saudi Arabia; 3 Production of bio-products for industrial application research group, King Abdulaziz University; 4 Department of Pharmaceutics and Industrial Pharmacy, Faculty of Pharmacy, King Abdulaziz University, Jeddah, Kingdom of Saudi Arabia; 5 Department of Pharmaceutics and Industrial Pharmacy, Faculty of Pharmacy, Al-Azhar University, Cairo, Egypt; 6 Biochemistry Department, Faculty of science, King Abdulaziz University, Jeddah, Kingdom of Saudi Arabia

**Keywords:** Review, *Leishmaniasis* prevalence, control, Saudi Arabia

## Abstract

**Background:**

*Leishmaniasis* is a widespread skin protozoan infectious disease. It is an intracellular parasitic microorganism that develops in the body of infected female phlebotomine sandflies vector, prior to its transmission to human or animal host by the vector bite. The objective of this review is to highlight the current prevalence of *Leishmaniasis* in the Kingdom of Saudi Arabia, and the direction in research for its control.

**Materials:**

The update literature covered The infection of the host with this trypanosome starts with a skin bite from the infected sand fly, followed by penetration of the parasite into cellular structures of the skin, or its infiltration to the circulatory system, targeting the internal organs. Different research groups are experimenting on construction of recombinant *Leishmania* antigens, compiled from this protozoa and from antigens recovered from the saliva of sand flies, in an attempt to immunize the host for protection against this disease.

**Conclusion:**

The benefits behind such a review is to support the personnel involved in developing evidence-based policy guidelines, strategies and standards for disease prevention and management of their implementation; in addition, it provided a technical support to member states to collaborate on establishment of an effective systems to handle the *Leishmaniasis*.

## Introduction

*Leishmaniasis* is a geographically widespread skin protozoan infectious disease^1^. It includes three main forms namely, Mucocutaneous leishmaniasis (ML), Visceral *leishmaniasis* (VL) and Cutaneous *leishmaniasis* (CL)^2^. There are more than 350 million people, in around 80 countries, that are at risk from this disease^4^, resulting in annual mortality of around 80,000 individuals^3^. *Leishmania* are infective protozoan pathogens, causing injury to the skin and/or internal organs. The etiology of the disease belongs to trypanosomes of the genus Leishmania, under order of Trypanosomatida, class of Kinetoplastea, and phylum of Euglenozoa. The pathogenicity in animal or human hosts of this parasitic etiology is characterized by its intracellular infectivity, following a bite from an infected sand fly. In the Old World, *Leishmaniasis* was caused by *Leishmania major* (*L. major*), *Leishmania tropica* (*L. tropica*) and *Leishmania aetiopica* (*L. aetiopica*), in which 90% of cases occurred in Afghanistan, Algeria, Iran, Iraq, Saudi Arabia and Egypt^5^. This disease is not common in other areas of the world, such as South East Asia. In Saudi Arabia, the disease is more prevalent in the Al-Hasa Oasis (Eastern Province), affecting evenly both human genders (Table 1). Authors: Please note that Table 1 doesn't show the equal frequency in both genders of the Estern Province. Among the three forms of *Leishmaniasis*, CL is predominantly spread, following a seasonal distribution, determined by higher activity of the sand fly^6^. CL varies in its clinical presentation. The mild dermal condition is usually self-healing; however, the resulting scars could be extremely disfiguring in appearance, leading to social and psychological stigma^7^. CL initial stage appears in a form of skin papule, developing later into a painless ulcerated and crusted nodule. The histopathology of the skin lesion shows a progression from a sheet of amastigote-filled histiocytes to a granulomatous inflammation stage. The manifestations of CL lesions vary, and could develop into other inflammatory complications, including neoplastic diseases^8^ CL was described for the first time in Kingdom of Saudi Arabia (KSA), by. Since that time, KSA kept reporting the presence of CL in its population; actually, the KSA is among the top 10 endemic countries in occurrence of CL; in 1996, the reports from KSA and five other countries showed a contribution in occurrence of CL equivalent to 90% of the global human cases^9^. *Leishmaniasis* could lead to a group of syndromes, affecting mostly the poor communities in developing countries, which could be the reason for the pharmaceutical industries not to invest in research to control this disease^10^. It is worth noting that 0.7 to 1.2 million new human cases of CL occur each year worldwide^11^.

**Table 1:** Parasitological information of Leishmaniasis available in Saudi Arabia

*Leishmania* species	Clinical form	Vector species	Reservoirs
*L. tropica*	CL	*P. sergenti*	
*L. infantum*	ZVL	unknown	*Canis familiaris*
*L. major*	ZCL	*P. papatasi*	*Meriones spp., Psammomys obesus*
*L. donovani*	VL	unknown	

*Leishmaniasis* is transmitted by the bite of infected female phlebotomine sandflies .The disease affects usualy the poorest people on earth, and is associated with malnutrition, population displacement, poor housing, weak immune system, and lack of financial resources^12^. *Leishmaniasis* is linked to environmental changes such as deforestation, building of dams, irrigation schemes, and urbanization. Despite of the great efforts by health authorities in KSA, CL continues to be a major public health problem in the country^13^. It is an endemic disease of Saudi-Arabia. Nonimmune Europeans and their families living temporarily in the endemic areas of the KSA are also affected by the disease. Many risk factors are contributing to the persistence of outbreaks and epidemics of CL in KSA; these most important factors are the rapid urbanization and the huge population movements to new areas in the country^14^. The disease is prevalent in five areas of the KSA namely, Al-Qaseem, Riyadh, Aseer, Ha'il, and Al-Madinah. *Phlebotomus papatasi* (vector of *L. major*) and Phlebotomus sergenti (vector of *L. tropica*) are the known vectors of the disease^15^. The confirmation of the diagnosis of CL is performed histologically, by identification of the presence of amastigote-life cycle stage in the host vertebrate, which varies significantly, depending on the dose in the inoculum, strain type, host response, and disease stage^16^.

Thus, CL continues to be a major public health problem affecting the communities in this country, and presenting a challenge to the national health authorities. The cell-mediated immunity is responsible for the skin lesion healing; however, humoral response plays also a protective role against the disease. Skin biopsies from 65 parasitological confirmed CL patients from Egypt, Saudi Arabia, Jordan and Libya were subjected to histopathology investigation. It was reported that CL, especially in hot areas, predisposes the skin cell genome to mutation, leading to skin neoplasm. It is documented that cases of CL, associated with caseating granulomas, showed a slower healing process; this slower process was not related to specific species of leishmania, but was hypothesized to be due to suppressed host immune response and/or strains of certain species with higher pathogenicity^17^. This review aimed at highlighting the current status of *Leishmaniasis* in KSA.

## Structure and life cycle

*Leishmania* can be represented by two forms - intracellular amastigota and promastigot^18^. Amastigot has rounded outlines, from 2.5 to 5 µm in diameter, located in the middle of the parasitophore vacuole of the macrophage. A clearly marked nucleus and kinetonucleus are observed, characterized by a vacuolated cytoplasm and the presence of lysosomes. The outer membrane contains a polysaccharide component, but without a glycocalyx layer. Promastigota is the presence of a clearly expressed flagella. The outer membrane contains binding molecules like glycoproteins and special cells of the immune system - manosis receptors. All this plays a big role in penetrating into the macrophage. This process is facilitated by the binding of plasma antibodies to promastigot.

*Leishmania* are located in the cell protoplasm of internal organs - it can be liver, kidneys, lungs, spleen, as well as cutaneous and mucous membranes, capillaries, etc. The affected cell can contain from one to two hundred leishmanias. *Leishmania* are representatives of trypanosomatides, which means their belonging to obligate parasites. The life cycle of *Leishmania* is determined by the presence of two successive hosts: an insect and a vertebrate^19^. Infection of insects with *Leishmania* occurs when they suck the blood of the carrier animal. Parasites with blood fall into the body of digestion of an insect: while in the middle intestine along the perimeter of the swallowed blood, an insect forms the so-called peritrophic matrix. Promastigotnaya form of the parasite reproduces in the digestive organs of female insects. Approximately 7 days later, infectious disease reaches the upper part of the digestive system. In this case, the *Leishmania* completely cover the digestive organ of the female. When an insect produces a bite to a mammal, its saliva, along with parasite accumulations, penetrates into the site of the bite into the skin of the new host.

As a rule, neutrophils - immune blood cells that capture parasites - tend to the site of damage. Within the cells, parasites exist until the time of natural death of neutrophils arrives. After this, *Leishmania* is released and unhindered in the blood of a mammal. Established within the human body or in the body of other mammals, *Leishmania* can be localized in the bloodstream and in the outer covers. Mosquitoes sucking out the blood particles of a diseased animal or human, are affected by *Leishmania*. Already on the first day, the swallowed parasite is transformed into a mobile flagellate form. It passes into the stage of reproduction and approximately in a week in the form of clusters it appears in the upper parts of the digestive tract of an insect^20^. With the bite of the affected insect, the active *Leishmania* penetrate into the microscopic wound, and thence into the cellular structures of the skin, or with the blood flow to the internal organs: this depends on the type of *Leishmania*.

## Various forms of *Leishmaniasis*

### Cutaneous form

The Cutaneous form of this disease usually cause skin ulcers on the visible parts of the body such as the arms, face as well as legs. It could cause a big number of lesions - sometimes up to two hundreds - causing serious invariably and disability leaving the patient lastingly scarred, a stigma that can cause serious social prejudice.

### Mucocutaneous form

In the form of mucocutaneous *leishmaniasis*, lesions could lead to total or partial destruction of the membranes of mucous of the nose, mouth in addition to the throat cavities and surrounding tissues. This degrading disabling form of *leishmaniasis* could lead in victims being humiliated as well as cast out from society. ML was diagnosed in a Filipino man who had worked in Saudi Arabia for 2 years. Two primary lesions-one on the forearm and one on the abdomen-were characterized by “satellite” papules and subcutaneous nodules extending proximally in a sporotrichoid pattern. *Leishmania* organisms were found in both primary lesions and a subcutaneous nodule. There was no evidence of systemic involvement^21^.

### Visceral form

VL (also known as kala azar) is widely seen form of *leishmaniasis*. Ii is considered by substantial loss in weight, bouts of fever irregularly, swelling of the liver and spleen and anaemia. If this disease left untreated, then the rate of fatality in the endemic countries could be spread rapidly.

## Prevalence

**Figure 2:**
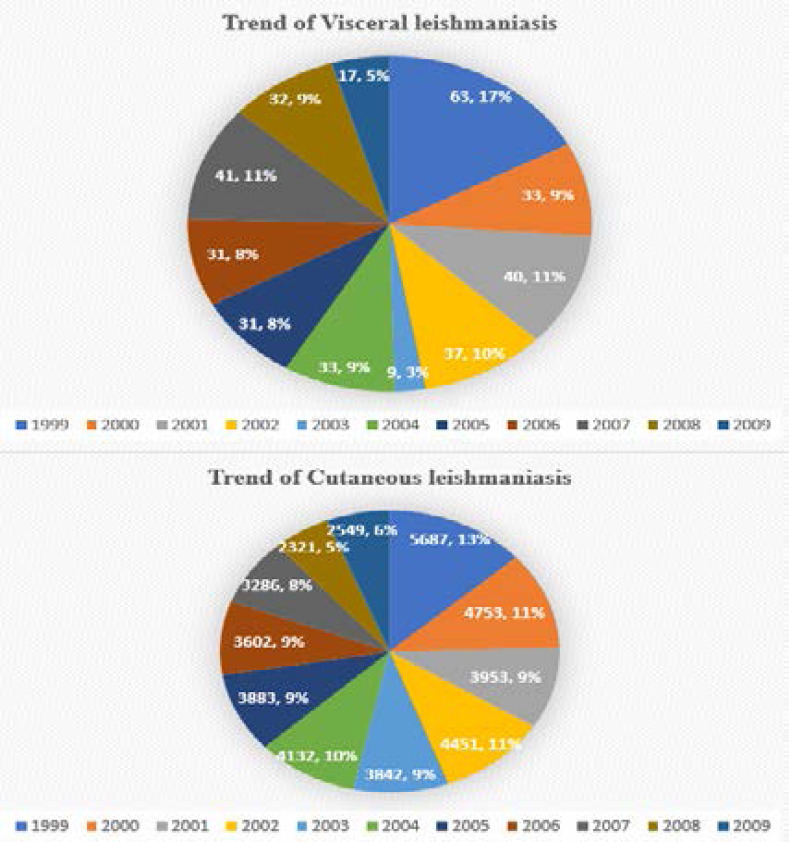
Trends of Visceral Leishmaniasis and Cutaneous Leishmaniasis in different regions of Saudi Arabia

VL is sporadic and mostly seen in Jazan region. In 2008, 29 of 32 cases reported in Saudi Arabia occurred here. 69% of cases were among young, male children as shown in fig (2). Cases of *Leishmaniasis* recidivans are not uncommon in the area^22^.

## Epidemic and Pandemic

### Route of infections

The VL is usually transmitted by the hematophagous sand-fly belonging to species Phlebotomus. However, The other transmission routs by 70 animals including dogs and man. It was reported that, blood transfusion and by other vectors such as ticks which may be included in the epidemiology of many diseases^23^. The transmission routes of infection contribute to the control and eradication strategies.

### The vector

*Leishmaniasis* are began by twenty different species pathogenic for humans going to the genus Leishmania, a protozoa transmitted by the bite of a tiny two to three millimetre-long insect vector, the phlebotomine sandfly. Within the *L. major* and *L. tropica* group, the subgroupings shaped had been mainly connected to the geographical origin of that particular strain (Shalaby^24^).

### Phlebotomine Sand fly

Only female Phlebotomine sand flies places their eggs in the holes of some rodents, in crumbling buildings, in the bark of old trees, in the cracks in walls of house, in shelters of animal and in household rubbish. Commonly in those environments that the larvae may find the heat and humidity, organic matter that are required for their development. The female sand fly infects itself and conveys the protozoa by the help of *Leishmania* parasites available in the blood it pulls from its mammalian or human host for obtaining the protein required to develop its eggs^25^. Throughout the period of four to twenty five days, these parasites start their development within the sand fly where they undergo a key transformation .When the new infectious female sand fly gets on a new blood source, its painful stings inoculate its new victim with the parasite. Thus, transmission cycle is completed. Phlebotomine sand fly, the insect vector of *Leishmaniasis* has been found through the inter-tropical and temperate regions of the world^26^. In its search for blood (usually in the evening and at night), the female sand fly covers a radius of a few to several hundred meters around its habitat.

### Sand cat

Sand cat or Felis margarita is a carnivore found in of the desert in many African and Arabian countries (sandy area). Ten species of sand cats were defined in Riyadh region. The host of sand cats as zoonotic for transmission of *Leishmaniasis* was detected in Saudi Arabia^27^.

## Mechanism of antigenic variation

The main variations in CL pathology in various geographical areas are most likely due to the differences between the species of *Leishmania* involved. However, not only do slight variations exist between patients from the same place, but they may also occur over time in the same patient. Therefore, it is difficult to compare areas in detail; lesions on one patient can heal asynchronously and display differences. histological types from the same lesion during healing reveals changes from one histological type to another.

## Impact on body

People having clinical sign of infection have one or more sores on their skin. These sores could change in the size and appearance followed by time. These sores may be beginning out as papules/bumps or nodules/lumps and may end up as ulcers, skin ulcers may be shielded by crust or scab. The sores usually are painless but painful. Few individuals have swollen glands near the sores (for example, under the arm, if the sores are on the arm or hand). Some people have a silent infection, without any symptoms or signs. People who grow clinical indication of infection typically have weight loss, fever, enlargement (swelling) of the liver, spleen and abnormal blood tests^28^. *Leishmaniasis,* which exists in both visceral and cutaneous forms, affect similar cells of the immune system like HIV.

## Control and prevention

The absence of a vaccine or chemoprophylaxis limits the options for leishmaniasis prevention.^29^ Elimination of reservoir species and some form of vector control, including barriers to sand fly feeding, are the primary instruments available. A reservoir population within a 500-m radius of a protected area should be removed in order to dramatically reduce disease risk^30^. In many environments, the complexity of achieving such control makes the vector control choice more appealing. In certain ways, management of the phlebotomine vector is very close to that used for malaria; however, most mosquito larval control strategies are inadequate for sand fly control since the mosquito larva's aqueous environment is very different from the highly organic soil requirement of sand flay. The use of Bacillus sphaericus for sand fly larval control is a potential exception. Bait-fed adults were used in this novel technique to bring the bacterial control agent to larval habitats in animal burrows, resulting in larval mortality in burrows up to 10–30 m away from the baited solution. However, the adult sand fly and the adult mosquito vectors share features that make control. In fact, some researchers suggest that a decline in the use of insecticides for malaria control is one explanation for the resurgence of *leishmaniasis*^31^. The majority of adult sand fly control spray techniques can be placed in the following categories:

## Conclusion and recommendations

Low awareness for important epidemiological aspects like transmission of the disease, risk factors and prevention are widely seen. This review provide a baseline to understand information regarding leishmaniasis in Saudi Arabia and would provide a template to design interventions. Supporting the national *leishmaniasis* control programs to produce updated guidelines and make disease control plans, raising awareness and advocacy on the burden of Saudi population suffering *leishmaniasis* this analysis will be productive.

## Figures and Tables

**Figure 1:**
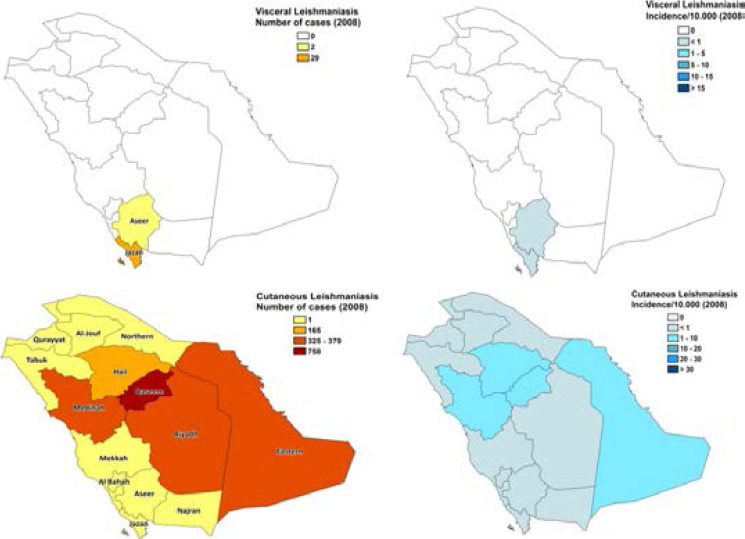
Maps of Visceral Leishmaniasis and Cutaneous Leishmaniasis in different regions of Saudi Arabia
